# Field assessment of insecticide dusting and bait station treatment impact against rodent flea and house flea species in the Madagascar plague context

**DOI:** 10.1371/journal.pntd.0007604

**Published:** 2019-08-06

**Authors:** Adélaïde Miarinjara, Soanandrasana Rahelinirina, Nadia Lova Razafimahatratra, Romain Girod, Minoarisoa Rajerison, Sebastien Boyer

**Affiliations:** 1 Medical Entomology Unit, Institut Pasteur de Madagascar, Antananarivo, Madagascar; 2 Ecole Doctorale Sciences de la Vie et de l’Environnement, Université d’Antananarivo, Antananarivo, Madagascar; 3 Plague Unit, Institut Pasteur de Madagascar, Antananarivo, Madagascar; 4 Department of Animal Biology, University of Antananarivo, Antananarivo, Madagascar; Instituto de Pesquisas Veterinarias Desiderio Finamor, BRAZIL

## Abstract

Bubonic is the most prevalent plague form in Madagascar. Indoor ground application of insecticide dust is the conventional method used to control potentially infected rodent fleas that transmit the plague bacterium from rodents to humans. The use of bait stations is an alternative approach for vector control during plague epidemics, as well as a preventive control method during non-epidemic seasons. Bait stations have many advantages, principally by reducing the amount of insecticide used, lowering the cost of the treatment and minimizing insecticide exposure in the environment. A previous study reported promising results on controlling simultaneously the reservoir and vectors, when slow-acting rodenticide was incorporated in bait stations called “Boîtes de Kartman”. However, little evidence of an effective control of the fleas prior to the elimination of rodents was found. In this study, we evaluated bait stations containing insecticide powder and non-toxic attractive rodent bait for their potential to control rat fleas. Its efficacy was compared to the standard method. The impact of both methods on indoor and outdoor rodent fleas, as well as the human household flea *Pulex irritans* were analyzed at different time points after treatments. Bait stations did not cause any significant immediate or delayed reduction of rat fleas and increasing the number of operational bait stations per household did not significantly improve their efficacy. Insecticide ground dusting appeared to be the most efficient method to control indoor rat fleas. Both methods appeared to have little impact on the density of outdoor rat fleas and human fleas. These results demonstrate limited effectiveness for bait stations and encourage the maintenance of insecticide dusting as a first-line control strategy in case of epidemic emergence of plague, when immediate effect on rodent fleas is needed. Recommendations are given to improve the efficacy of the bait station method.

## Author summary

Insecticide ground dusting inside houses is the recommended measure to control rat fleas responsible for bubonic plague transmission. The main inconvenience of this method is the direct contact of houseowners to the toxic insecticide dust and spillage in environment. A bait station approach, where the insecticide is confined in a box or tunnel containing rodent attractant, seems to be a valuable complementary or alternative vector control tool. However currently, little is known about its real efficacy on reducing or eliminating fleas harbored by rats. Guidelines regarding its implementation (density and duration of use) as vector control tool are lacking. Those questions were addressed during a field trial study, where bait stations were deployed at different densities per household and followed up at different time points. The efficacy of bait station was compared to the standard method. The present study allowed to demonstrate that bait station approach requires more improvements to be efficient. Meanwhile, insecticide ground dusting is still recommended for to control rat fleas during epidemics.

## Introduction

The *Yersinia pestis* bacterium, the causative agent of plague, is transmitted between rodents by infected flea bites [1]. The plague cycle involves wild and commensal rodents and their fleas; humans are accidental hosts. This zoonotic disease can lead to significant mortality amongst susceptible rodents, thus releasing host-seeking potentially infected fleas. Humans can develop the bubonic form of plague, when the bacterium is deposited in the dermis by fleas [2]. The bubonic form is characterized by fever, headache, chills, and painful inflammation of lymph nodes draining the site of the flea bite. *Yersinia pestis* can disseminate from the lymph nodes into the bloodstream causing septicemic plague. Secondary pneumonia occurs when the infection reaches the lungs [2]. Pneumonic plague is responsible for inter-human transmission by inhalation of contaminated droplets and can be associated with high mortality rate if inadequately treated. The disease can be cured, if diagnosed early and treated with the adequate antibiotic. Poor housing and hygiene, proximity to rodents and fleas in everyday life are major and unchanged risk factors in both historical and modern episodes of plague [3]. Currently, most infections of human plague have been reported in low-income countries in the African region. Madagascar has become the country with the highest plague burden [4]. Between 1998 and 2016, 250 to 1500 suspected cases were reported annually by the Malagasy Central Laboratory for Plague, with 18% case-fatality rates for confirmed and presumptive case-patients (1,057/5,819) [5]. The bubonic plague is the most encountered form (about 80%), however, secondary pneumonic plague can lead to overwhelming urban epidemic, such as reported in the capital in 2017 [6,7].

In the Central Highlands of Madagascar, plague epidemics mainly occur in remote rural areas, with small household epidemic patterns, during the rainy and warm months from October to April [8]. In rural settlements, housing is usually constructed of mud with thatched roof and the floor is covered with a braided vegetal fiber mat. This type of housing is easily accessible by *Rattus rattus*, the main reservoir. Traditional outdoor granaries are infrequently used due to thief problems. Crops and food are stored in the bedroom, which promotes contact between humans, rodents and their fleas [5,8]. Two flea species have been reported as confirmed vectors. *Xenopsylla cheopis*, the oriental rat flea is considered the main vector, parasitizing *R*. *rattus*, *R*. *norvegicus*, and *Suncus murinus* in rural and urban areas [8]. *X*. *cheopis* is associated with indoor rats, which makes this species the target of vector control during plague episodes. *Synopsyllus fonquerniei* is an endemic flea vector, found mainly in rural areas, above 800 meters of elevation. This species is associated with outdoor *R*. *rattus* but can also be found in forest areas harbored by small endemic mammals [8]. Recently, *X*. *brasiliensis* was described for the first time in the northern plague focus [9]. The human flea, *Pulex irritans*, a species that is not associated with rodents, is the most abundant species caught inside human houses. It has also been found naturally infected with plague bacterium during an epidemic [10]. Further studies are needed to determine its involvement in the plague cycle and its competence as a vector.

In the context of public health, flea control can play an important role in preventing the expansion of plague epidemic. Reducing the density of rodent fleas as fast as possible is the main purpose of all techniques developed for flea control [11]. Insecticide dusting is the main method recommended by WHO to control rat fleas during plague outbreaks [12–14]. It is now recognized that flea control must be performed before rodent control, in order to minimize the spread of infected fleas and the risk of transmission of the bacterium to humans [11,13]. Insecticide dust may be blown into the entrance of rodent burrows but more often a layer of insecticide is deposited on rodent runways, usually indoors at the bottom of the walls [13]. This insecticide treatment is not always accepted, because of toxicity and concerns for health and environmental side effects [15]. The use of host-targeted methods such as a “bait station” or “bait box” appeared to reduce the risks of insecticide exposure, mainly for non-target species and children [12]. In addition, this method can reduce the cost of treatment, because less insecticide is required [16]. Insecticide powder is placed inside a container with a bait to attract the rodents hosting targeted fleas. Rodents entering the bait station come in contact with the insecticide. The bait station technique was found to be highly effective to target ectoparasites of wild rodents [16–21]. However, its use to prevent human plague expansion has been less investigated. Yet, a bait station approach may be a good alternative to direct insecticide dusting inside households.

In Madagascar, insecticide dusting inside homes is one of the measures recommended by the National Plague Program, to prevent the spread of plague epidemics [22]. Since *X*. *cheopis*, the main vector, is mainly harbored by indoor rodents and plague epidemics occur mostly during the rainy season, insecticide treatments are usually performed indoors. Bait stations have become popular and used as a complementary tool against rat fleas [23,24]. In 2003, a study conducted in the Malagasy capital, Antananarivo, aimed to evaluate the use of wooden tunnel bait stations (“Boîtes de Kartman”) containing bait incorporating anticoagulant rodenticide, to simultaneously control the reservoir and vectors of plague [23]. The results appeared promising, with significant reduction of rodent density, but it was difficult to prove the effectiveness of the method for flea control, especially before the control of targeted rodents. Furthermore, elimination of the rodent population during an epidemic may favor the immigration of non-treated and potentially infected rodents from the surrounding area [25]. The objective of this project was to evaluate the efficacy of bait station method on reducing rat flea density while maintaining the rodent population. The results at each time point were compared to the efficacy of direct ground dusting, which remains the standard method.

Rat movement between indoor and outdoor environments is well documented in Madagascar [26,27]. Insecticide treatments are focused indoors, while exposure to potentially infected small mammal harboring *S*. *fonquerniei* is possible outdoors. In this study the impact of both indoor insecticide treatments on outdoor rat flea prevalence was also evaluated.

Despite the conflicting role attributed to *P*. *irritans* during human plague epidemics [10], house fleas are not the principal target of vector control in Madagascar. A study conducted in Tanzania showed a correlation between *P*.*irritans* density and the risk of bubonic plague [28]. Yet the potential of indoor insecticide treatments on reducing *P*. *irritans* prevalence is investigated here for the first time.

To summarize the objectives of this project, the efficacy of bait station method and direct insecticide dusting treatments on reducing (1) indoor and (2) outdoor rat fleas, as well as (3) house flea burden will be evaluated. For each condition, the evaluation was performed over two days, six days, and one month after treatment.

The study was conducted in two stages in 2016 and 2017. Field experiments conducted in 2016 compared the effectiveness of direct insecticide dusting and bait station approach on reducing flea burden. Based on the results obtained in 2016, a subsequent study was implemented in 2017 and focused on examining if increasing the number of operational bait stations or using them for a longer period in households could improve the efficacy.

## Methods

### Ethics statement

The study protocol was approved by a local “Ad-Hoc” committee including the University, the Veterinarian School of Antananarivo, and the Ministry of livestock of the Malagasy Government (Institut Pasteur de Madagascar institutional committee for animal ethic) and rodent sampling was conducted in accordance with the directive 2010/63/EU of the European Parliament (http://eur-lex.europa.eu/Lex UriServ/LexUriServ.do?uri=OJ:L:2010:276:0033:0079:EN:PDF).

### Study site

**Fig 1:**
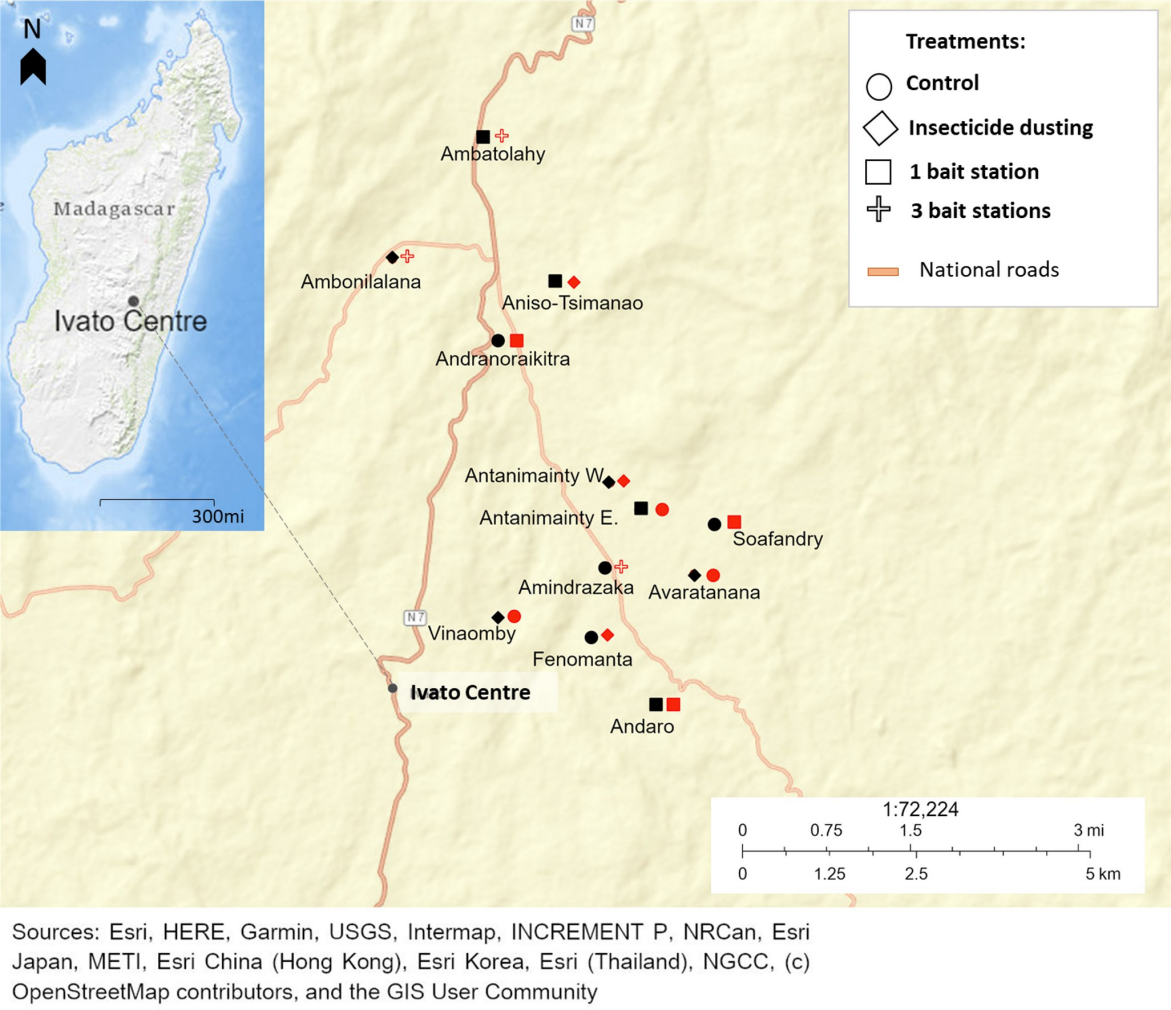
Map of study sites. The shapes represent the treatment groups assigned to each study site in 2016 (in black) and 2017 (in red). In the upper-left corner is a map of Madagascar showing the location of the commune Ivato Centre. Map services and data available from U.S. Geological Survey, National Geospatial Program: https://viewer.nationalmap.gov/advanced-viewer/.

The study was carried out in the commune of Ivato Centre, district of Ambositra, Madagascar (Fig 1). This district, located in the southern part of the Central Highlands, lies within the plague endemic region of Madagascar [29]. Field trials were conducted during the dry and cool season of low plague transmission. This timeframe corresponded to the end of the rice harvest season in the Central Highlands, which is linked to high rodent numbers in the household environment [27].

Twelve hamlets of about 20 houses each were selected for the study and assigned randomly to treatment groups (Fig 1). The closest hamlets were separated by approximately one kilometer to prevent rat immigration between hamlets [27]. Study sites were included depending on their accessibility, verbal consent and acceptance of the local leader and household head.

Hamlets included in the study were generally organized as described previously [27], with houses constructed on hill tops and rice fields located in the valleys below (S1A and S1B Fig). Housing was usually a three-level home (ground floor, first floor and the attic) with at least two rooms per level, constructed of brick or mud walls and a thatched roof (S1B and S1C Fig). Dirt floors covered with plant fiber mat, or wooden planks were the most common. Homes with concrete floors were rare. The main rooms were located on the first floor and the attic. The ground floor was generally intended for domestic animals (livestock, rabbits and poultry).

### Rodent and flea processing

The efficacy of each treatment on rodent fleas was evaluated by measuring the following parameters during pretreatment and post-treatment rodent sampling occasions: flea index (FI) and infestation rate (IR). For each group and at each sampling occasion, the flea index was defined as the mean number of fleas per animal and infestation rate as the percentage of small mammals harboring at least one flea.

Rodents were live-trapped using Sherman (HB Sherman Traps, Inc., Tallahassee, FL) and wire-mesh BTS (Besançon Technique Service, FR) baited with dried fish and onions. Trapping was conducted for one night in each hamlet and each sampling occasion. Captured animals were euthanized and brushed with fine hair brush over a pan to remove fleas. Body weight and morphological measurements such as head to body, tail, ear, and hind foot length were recorded to identify the small mammal species. Escaped and devoured (partially consumed by predator) rodents were removed from the data analysis. Fleas collected from rodents were stored in microcentrifuge tubes containing 95% ethanol for later species identification.

### Study design during 2016 field trials

The study involved three arms called “groups”, each involving four hamlets (Fig 1). Fenitrothion powder (2%, organophosphate. Prochimad, Antananarivo, Madagascar) was used in bait stations and dusting. For indoor trapping, a total of 20 Sherman traps and 20 wire-mesh traps were used per hamlet (one of each per house). For outdoor trapping 20 wire-mesh BTS traps per hamlet were deployed and distributed within 5 to 20 meters around the homes. Indoor and outdoor rodent sampling was performed over three sampling periods for each group.

The first rodent sampling was conducted, one day before treatment (day 0) to have initial values of parameters, and to assess the uniformity of pretreatment data collected in each group. A second rodent sampling was done in all groups two nights after insecticide dusting and the placement of bait stations, to evaluate the immediate effect (day 2) of each treatment. The third rodent trapping was completed one month after treatments (day 30) to measure the residual effect of each insecticide treatment.

Four hamlets did not receive insecticide treatment and considered as “control group”. Four hamlets, assigned as “bait station group”, had one bait-station per house, and bait stations remained for two consecutive nights within each house. Bait stations called “Boîtes de Kartman” were constructed as described in previous research [23,27]. The bait stations were locally made four-sided wooden tunnels, measuring 40 cm long x 10 cm wide x 10 cm high. The box was divided into three compartments, with non-poisoned rodent bait in the middle and insecticide dust layer in about 0.8 cm deep at both ends. Each bait station contained approximately 20 grams of manually rolled rat pellets (mixture of laboratory animal diet powder, wheat flour, peanut oil, and water). The upper side of the bait station is removable to facilitate baiting and deposition of insecticide (S2 Fig). Bait stations were placed indoors, on the floor, against the wall or on the top wall plate beneath the roof, according to the signs of rodent presence (droppings, holes, tracks).

Insecticide dusting was performed in four hamlets called “dusting group” with a hand shaker [12]. Insecticide dust was deposited on the indoor floor surface located 20 to 30 centimeters from the base of the wall, in a layer about three millimeters thick (50 grams per m²). Entries of rat and mouse burrows were also dusted. The rooms and kitchen were treated after the owner's consent (certain rooms were not allowed to be treated because of presence of newborn babies or domestic animals). Food, cooking utensils and other personal items were removed from the surface to be treated and homeowners were encouraged not to sweep away the insecticide during the study.

The impact of treatments on house fleas was monitored by collecting them in all groups, at the same time as rodent sampling. House fleas were trapped using a candle trap (candle placed in the middle of a plate containing soapy water) set in the middle of bedroom and lit by the homeowner when they turned off their own light. Fleas were attracted to the light and fell in the soapy water [30]. Trapped fleas were collected manually the next morning using fine forceps and preserved in 95% ethanol. All fleas were later identified at the species level under binocular microscope (40 x magnification).

Fleas collected from small mammals during day 30 sampling were transported alive to the laboratory to conduct insecticide susceptibility tests. Adults fleas were identified at species level and *X*. *cheopis* were reared under laboratory conditions until the next generation reached enough numbers to perform insecticidal bioassays as described previously [31]. Each batch of fleas was exposed to 1% fenitrothion impregnated filter paper (1.5 x 6 cm, Vector Control Research Unit, Penang, Malaysia) during the diagnostic period. Mortality was recorded 24 hours after exposure.

### Study design during 2017 field trials

The same hamlets (twenty houses each) as in the 2016 study design were included in 2017, but a new random treatment assignment at hamlet level was organized, independently of hamlet status in previous study (Fig 1). BTS and Sherman traps were deployed indoors only. The same bait stations as in the 2016 study were used. Instead of rolled pellet, ripe tomato slices were used as attractant. Baits consumption was checked daily and were replenished when necessary by locally recruited health care workers. Rodent sampling was performed before the treatment to collect baseline data (day 0), day two post-treatment (day 2) to assess the immediate effect, and six-day post-treatment (day 6) to evaluate the effect of longer usage of bait stations (day 2 *versus* day 6). No sampling was done at day 30 post-treatment.

Three hamlets were set as the “control group” and did not receive any insecticide treatment. Three hamlets received insecticide dusting following the similar protocol as described for 2016 study and were designated as the “dusting group”.

Three hamlets, assigned as the “one bait station group”, received one bait station per household over six consecutive nights.

The remaining three hamlets received three bait stations per household (one for each story) and were assigned as the “three bait station group”. Bait stations were left in house for two consecutive nights. Post-treatment sampling was done at day 2 only, to evaluate the immediate effect of increasing the number of deployed bait stations.

### Data analysis

The relative abundance of rats was calculated based on trapping success: number of animals captured divided by number of trap nights.

Flea index, flea range, and infestation rate were calculated per sampling date for all groups [32]. Uniformity of FI and IR obtained from each group before treatment (day 0) was tested using Kruskal-Wallis and Chi-square test, respectively. Differences were considered significant at p value < 0.05. Separate analyses were done for indoor and outdoor-collected rat fleas.

To demonstrate which treatment (insecticide dusting or bait station) was the most effective, post-treatment FIs recorded in each group were compared to control group using the Kruskal-Wallis test [32]. The infestation rate between groups was compared two by two, using a Chi-square proportion test [32]. The most effective method was assumed to be the one resulting in significant decrease of FI and IR post-treatment, compared to the control.

To compare the efficacy of each method over time, intra-group evaluations were conducted by comparing FIs between day 0, day 2 and day 6 or day 30, using the Kruskal-Wallis test followed by a Dunn test, a post-hoc analysis test for multiple comparisons. The same comparison was performed between IR values, using Chi-square proportion test. The efficacy over time for each insecticide treatment was proved by a significant reduction of FI and IR when compared to the initial values (day 0).

The percent control achieved by each treatment method at each time point was calculated using Henderson and Tilton‘s method [33,34]. The percent control of FI by a given treatment was determined by comparing the FI of treated group with the FI of the control group as follows: percent control = 100-(T/U x 100), where T = the post-treatment FI divided by the pre-treatment FI in the treated group and U = the post-treatment FI divided by the pre-treatment mean in the control group. The treatment was considered inefficient when the percent control value was below or equal to zero.

For house fleas, the mean flea number per house (total flea number per hamlet divided by the number of sampled houses) and range were recorded for groups at each sampling point. On day 0 the mean flea numbers (decimal logarithm transformed data) per hamlet were compared, using ANOVA test [35]. To evaluate the effectiveness of the method on house fleas, changes in flea number per house before and after treatment were analyzed separately for each hamlet. A two-way ANOVA test followed by Tukey test was used to compare the two sampling periods after treatment [35].

## Results

### August 2016 field study

#### Capture of rodents

We captured a total of 683 small mammals during the 2016 field study, with 31.2% overall trapping success. Four rats were found devoured in the traps and were removed from the dataset, giving a total of 679 rodents and shrews (Table 1). A total of 404 mammals belonging to three species was captured indoors: *R*. *rattus* (n = 252), *Mus musculus* (n = 149) and *S*. *murinus* (n = 3). Among the 275 small mammals caught during outdoor trapping, 99.0% were *R*. *rattus* and 1.0% *S*. *murinus*. The overall flea index, range infestation rate, and number of captured hosts per group and at each sampling day are presented in Table 1. A total of 274 fleas belonging to two species was collected during the study: mainly *X*. *cheopis* from indoor-trapped animals (95.7%) and *S*. *fonquerniei* from the outdoor captures (60.9%).

**Table 1:** Captured fleas and rodents during 2016 and 2017 studies.

		2016*					2017				
Groups	Sampling	FI	Range	IR (%)	Host number	Percent control	FI	Range	IR (%)	Host number	Percent control
Control	Day 0	0.58	0–8	26.10	69	NA	3.00	0–18	57.58	33	NA
	Day 2	0.54	0–7	23.80	63	NA	2.50	0–10	67.85	28	NA
	Day 6	_	_	_	_	NA	3.29	0–11	64.70	17	NA
	Day 30	0.71	0–9	30.90	68	NA	_	_	_	_	NA
Dusting	Day 0	0.49	0–4	25.00	83	NA	1.68	0–12	39.3	28	NA
	Day 2	0.15	0–4	10.20	88	67.12	0.33	0–3	29.17	24	76.43
	Day 6	_	_	_	_	_	0.29	0–2	23.53	17	84.26
	Day 30	0.66	0–6	27.70	70	-10.03	_	_	_	_	_
1 bait station	Day 0	1.02	0–9	34.40	96	NA	1.70	0–9	45.00	20	NA
	Day 2	0.68	0–11	24.70	73	28.40	1.47	0–8	53.33	15	-3.76
	Day 6	_	_	_	_	_	0.88	0–5	56.25	16	52.80
	Day 30	2.30	0–72	44.90	69	-84.20	_	_	_	_	_
3 bait stations	Day 0	_	_	_	_	_	1.54	0–23	33.30	15	NA
	Day 2	_	_	_	_	_	1.04	0–7	45.83	20	18.97

*For 2016, indoor and outdoor data are presented without segregation. FI: flea index, IR (%): infestation rate, NA: not appropriate, “_”: missing data, not determined during the study.

#### Effect of insecticide application methods on indoor rodent fleas.

**Fig 2:**
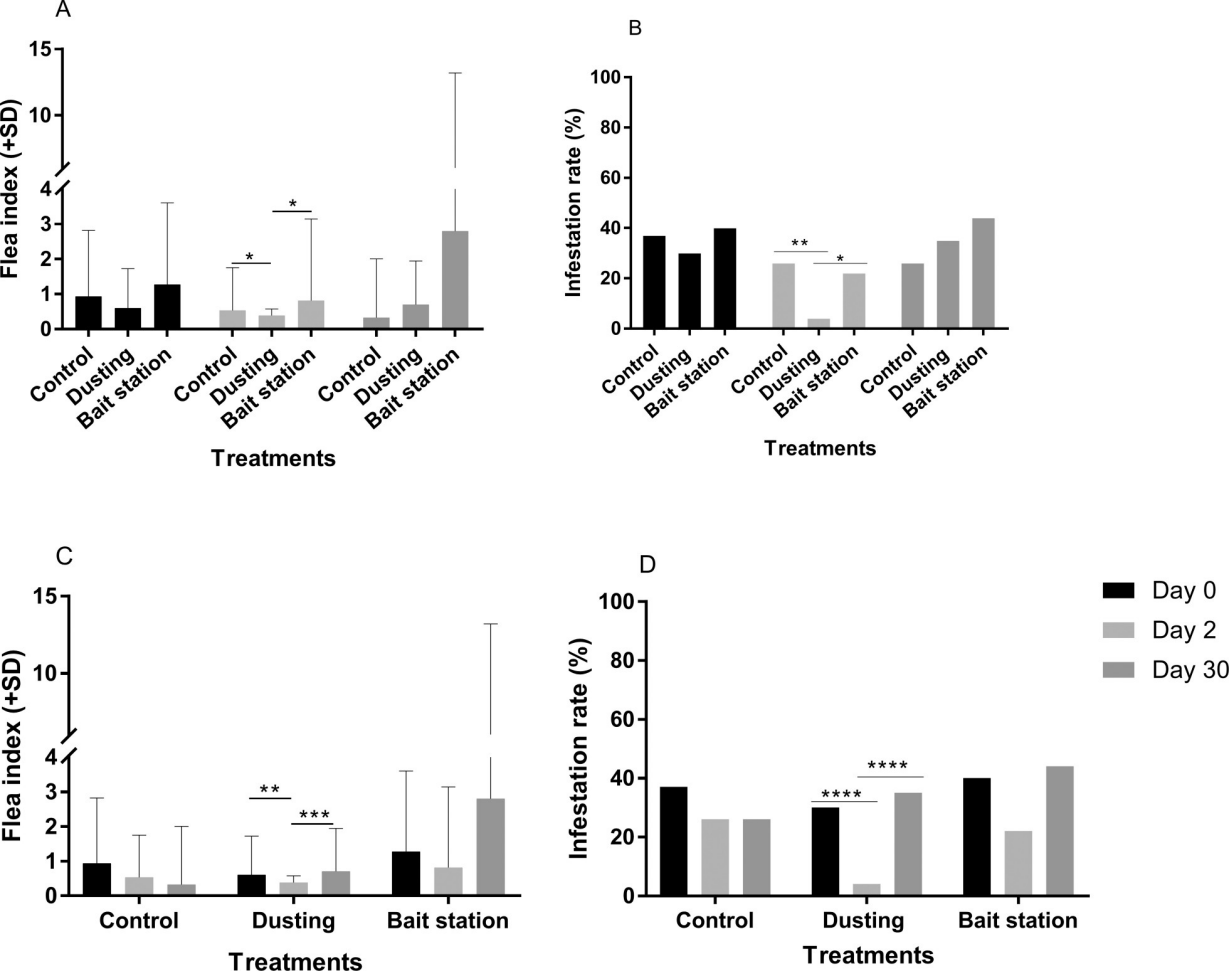
2016 study—Flea index and infestation rate for indoor rodents. (A) Comparison of flea indexes. (B) Comparison of infestation rates. At day 2, significantly lower flea index and infestation rates were observed for the dusting group when compared to control and bait station groups. (C) Efficacy of each method over time on reducing flea index. (D) Efficacy of each method over time on reducing infestation rate. In the dusting group, significantly lower flea index and infestation rates were observed on day 2 post-treatment when compared to day 0 and day 30 (*: p value<0.05, **: p value<0.01, ***: p value<0.001, ****: p value<0.0001).

During the initial rodent sampling (day 0), no significant difference between FI (Fig 2A) nor IR (Fig 2B) between groups was observed. At day 2, insecticide dusting was the most efficient method, with significant lower FI (Fig 2A) and IR (Fig 2B) in the dusting group. Bait stations did not significantly decrease the FI (Fig 2A) and IR (Fig 2B), compared to the control.

The immediate effect of insecticide dusting was observed on indoor rodents, two days after insecticide treatment, by a significant drop of FI (Fig 2C) and flea IR (Fig 2D), when compared to day 0. Only two rodents harboring one flea each were found in dusted hamlets two days after the insecticide application, corresponding to 67.1% control. However, one month later, those parameters were found equivalent to their initial values observed in the preliminary catch (Fig 2C & 2D) and the percent control calculation yielded negative values (Table 1).

Deployment of bait stations in households did not significantly affect the flea load on indoor rodents (Fig 2C & 2D). Two days after treatment, 28.4% control was recorded, and this value was below zero at day 30 (Table 1). In the control group, FI (Fig 2C) and IR (Fig 2D) did not vary significantly over time.

#### Effect of indoor insecticide application methods on outdoor rodent fleas

**Fig 3:**
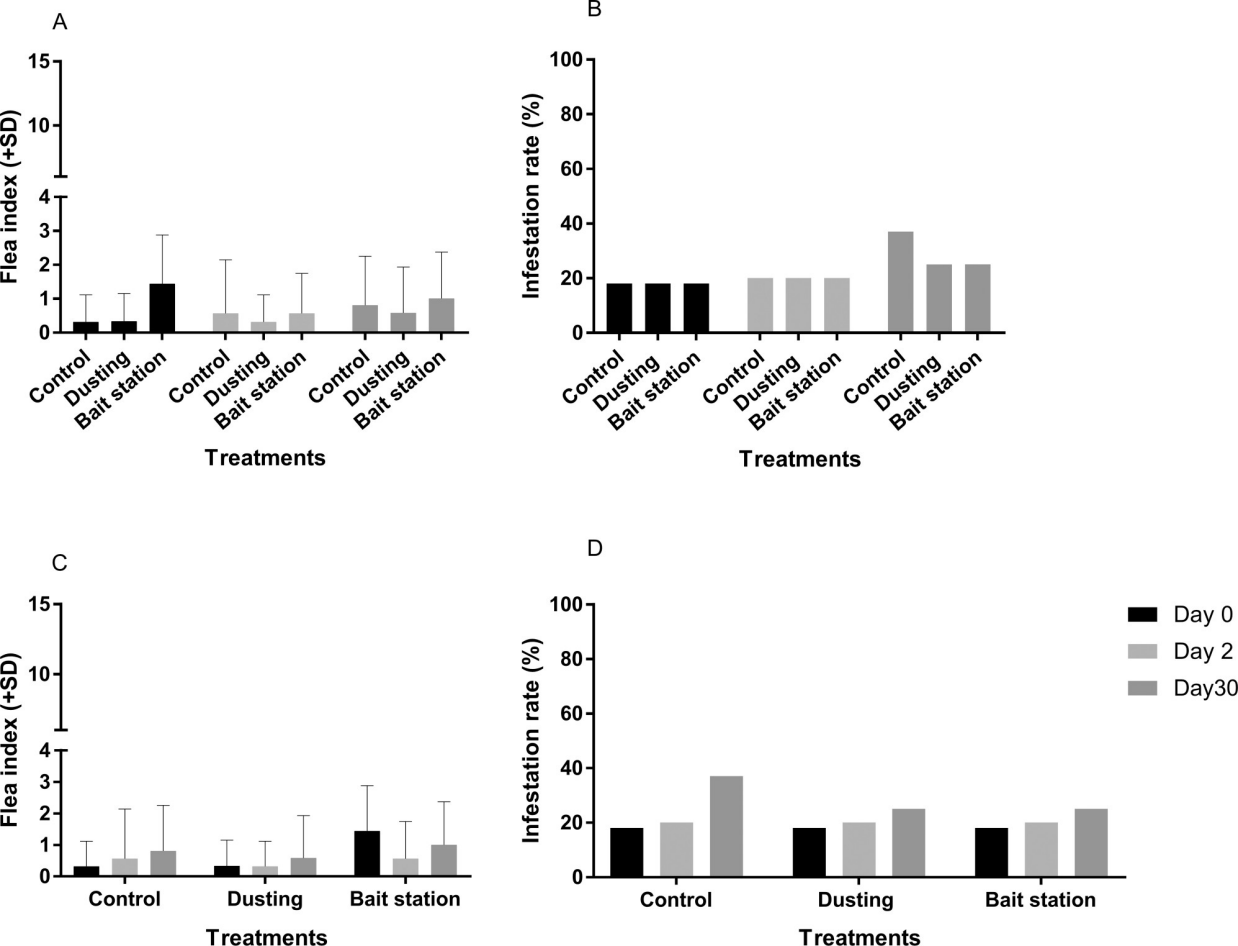
2016 study–. flea index and infestation rate for outdoor rodents. (A) Comparison of flea indexes. (B) Comparison of infestation rates. (C) Efficacy of each method over time on reducing flea index. (D) Efficacy of each method over time on reducing infestation rate.

No significant differences were observed between values of FI, as well as IR, on outdoor-caught rodents at each time point and between groups (Fig 3C & 3D).

#### Effect of insecticide application methods on house fleas

A total of 23,220 fleas were collected with the candle trap during the August 2016 study. *Pulex irritans* represented 99.0% of overall collected fleas, contrary to fleas collected from rodents, followed by *Ctenocephalides felis* (n = 167). One *X*. *cheopis* and one *S*. *fonquerniei* were also collected. Flea numbers caught per house in one night was within a range of 0 to 534, meaning that house flea distribution was very heterogeneous amongst houses in the same hamlet (Table 2).

**Table 2:** Collected house fleas during 2016 study.

Hamlet	Sampling date	Treatment	Mean flea number	Standard deviation	Flea count	Minimum	Median	Maximum
Amindrazaka	D0	Control	26.15	25.96	523	0	20.0	111
Amindrazaka	D2	Control	23.25	27.62	465	0	15.5	111
Amindrazaka	D30	Control	31.15	40.31	623	0	12.5	160
Andranoraikitra	D0	Control	45.10	85.54	902	0	10.5	352
Andranoraikitra	D2	Control	27.10	27.34	542	0	14.0	102
Andranoraikitra	D30	Control	24.85	38.32	497	0	17.0	175
Fenomata	D0	Control	20.45	20.65	409	3	13.0	86
Fenomata	D2	Control	35.55	44.39	711	0	24.5	184
Fenomata	D30	Control	42.85	79.81	857	0	7.5	353
Soafandry	D0	Control	11.30	12.36	226	0	7.5	50
Soafandry	D2	Control	9.60	15.54	192	0	3.5	65
Soafandry	D30	Control	17.55	30.42	351	1	8.0	134
Ambatolahy	D0	Bait station	29.00	27.10	580	0	24.5	88
Ambatolahy	D2	Bait station	30.15	39.56	603	0	12.0	145
Ambatolahy	D30	Bait station	26.85	31.26	537	0	14.5	128
Andaro	D0	Bait station	76.05	103.76	1521	0	37.5	324
Andaro	D2	Bait station	67.32	76.00	1279	2	34.0	298
Andaro	D30	Bait station	32.26	29.42	613	0	21.0	108
Aniso-Tsimanao	D0	Bait station	38.10	38.94	762	0	26.5	110
Aniso-Tsimanao	D2	Bait station	28.85	28.17	577	0	23.0	99
Aniso-Tsimanao	D30	Bait station	29.45	26.04	589	0	22.0	105
Antanimainty E.	D0	Bait station	17.75	14.97	355	0	15.5	49
Antanimainty E.	D2	Bait station	20.44	26.25	368	0	11.5	104
Antanimainty E.	D30	Bait station	25.28	21.59	455	2	18.0	80
Ambonilalana	D0	Dusting	66.60	102.16	1332	0	32.0	460
Ambonilalana	D2	Dusting	75.10	117.99	1502	0	41.5	534
**Ambonilalana**	D30	Dusting	23.30	25.76	466	0	15.0	95
Antanimainty W.	D0	Dusting	32.50	26.85	650	0	24.5	107
Antanimainty W.	D2	Dusting	39.35	28.72	787	0	33.5	104
**Antanimainty W.**	D30	Dusting	13.55	13.26	271	0	10.0	39
Avaratànana	D0	Dusting	33.37	59.54	634	0	7.0	221
Avaratànana	D2	Dusting	26.53	57.08	451	1	8.0	241
Avaratànana	D30	Dusting	32.63	43.71	522	0	19.0	176
Vinaomby	D0	Dusting	38.25	45.49	765	1	26.5	196
Vinaomby	D2	Dusting	31.65	28.69	633	0	25.5	108
Vinaomby	D30	Dusting	33.50	30.30	670	0	27.5	119

Bold characters: hamlets where significant decrease of mean house flea was recorded at day 30, when compared to day 2.

**Fig 4:**
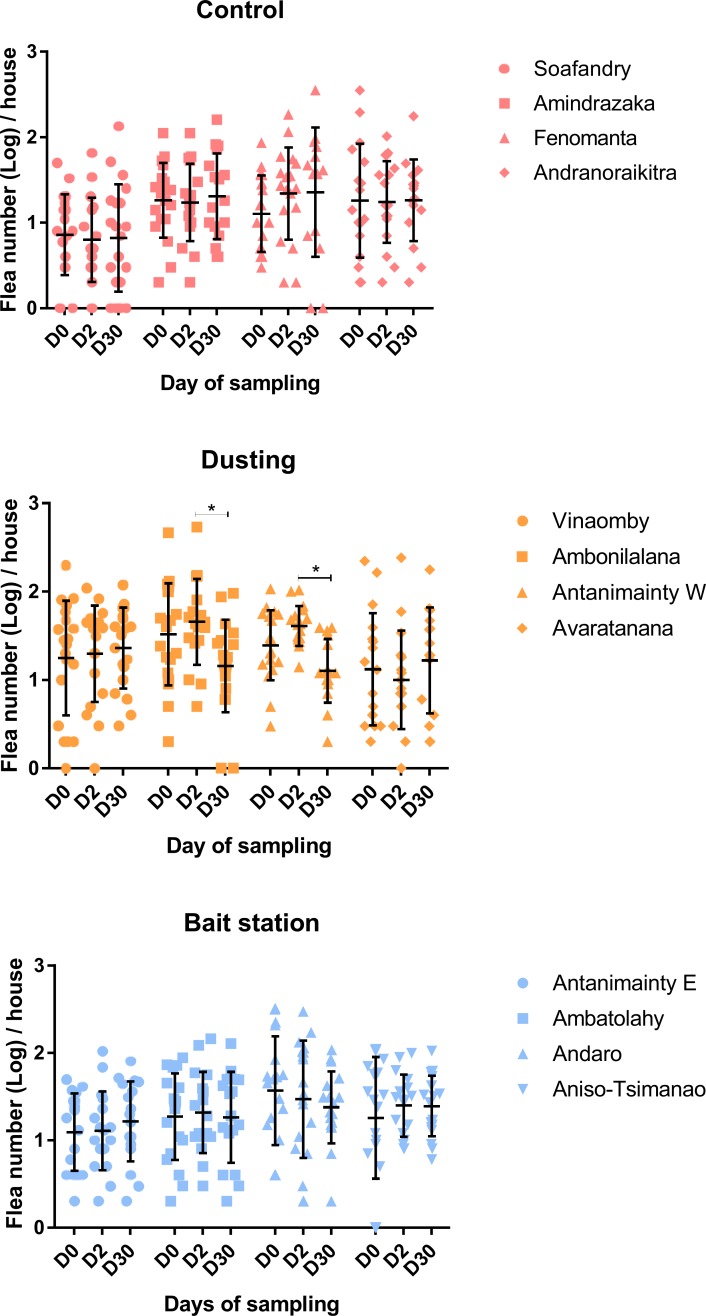
House flea number per hamlet. Each dot represents the number of fleas per house. The horizontal bar represents the mean number of fleas per hamlet and error bars are the standard deviations. A significant decrease in average flea number per house was observed at day 30 in two hamlets belonging to the dusting group, when compared to day 2 post-treatment *: p value < 0.05.

During initial capture, mean flea numbers per house were significantly different among the 12 hamlets (p = 0.021) and there was a strong hamlet effect. Therefore, the effect of both insecticide treatments was assessed at hamlet level. In the control and bait station groups, there were no significant differences in the mean flea number per house between day 0, day 2, and day 30. However, in two hamlets belonging to dusting group, significant decreases in mean house flea number per household were observed at day 30, when compared to day 2 after insecticide treatment (Fig 4).

#### Insecticide susceptibility tests

Laboratory insecticide tests of rodent fleas showed total susceptibility to fenitrothion, with a mortality rate of 100% at the end of the 24-hour observation period.

### August 2017 field study

#### Capture of rodents

A total of 246 small mammals were caught during August 2017, of which 175 were *R*. *rattus*, 68 *M*. *musculus*, and 3 *S*. *murinus*. Ninety-seven percent of fleas caught on rodents were *X*. *cheopis*. Details about flea index, flea infestation, and rodent number per treatment group and at each sampling occasion are given in Table 1.

#### Effect of insecticide application methods on indoor rodent fleas

**Fig 5:**
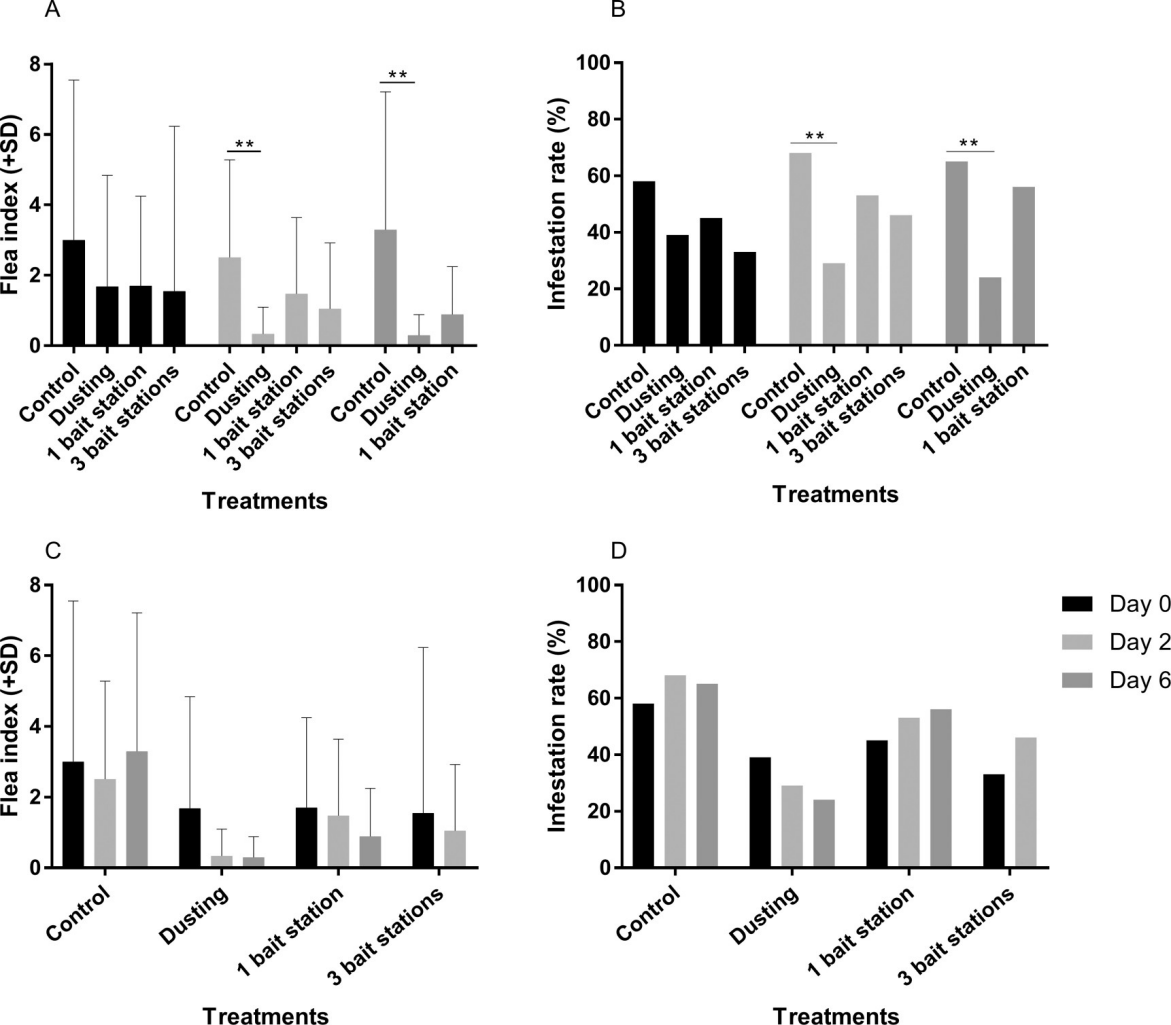
2017 study—Flea index and infestation rate for indoor rodents. (A) Comparison of flea indexes. (B) Comparison of infestation rates. At day 2, significantly lower flea index and infestation rates were observed for the dusting group when compared to control and bait station groups. (C) Efficacy of each method over time on reducing flea index. (D) Efficacy of each method over time on reducing infestation rate (**: p value<0.01).

Before treatment, flea indexes and infestation rates were the same in all groups (Fig 5A). Insecticide dusting appeared the most efficient method to control rodent fleas, as demonstrated by lower flea index and infestation rate observed at day 2 and day 6 post-treatment (Fig 5A & 5B).

The group with one bait station per house had no significant difference of FI and IR between day 2 and day 6 (Fig 5C & 5D). However, 52.8% control was observed at day 6, while this value was below zero at day 2 (Table 1). This implies that using one bait station longer than two days did not improve efficacy. Post-treatment values were the same as on day 0.

In the three-bait station group, no immediate effect was observed two days post-treatment (Fig 5C & 5D). Flea indexes and Infestation rate were the same for the one bait station and three bait station groups at day 2 (Fig 5C & 5D). Increasing the number of available bait stations did not significantly improve the impact of bait station method, even if 18.9% control was recorded in three bait station group, two days after the bait station set up (Table 1).

## Discussion

### Effectiveness of insecticide dusting

Fenitrothion powder (2%), used as ground dusting treatment was effective in reducing flea load on rodents 48 hours after the completion of the treatment. This result is in accordance with the emergency context of plague outbreak, as exposure for 24 h should be sufficient to kill adult *X*. *cheopis* on rats [13]. In addition, insecticide susceptibility tests showed great susceptibility to fenitrothion and is consistent with previous studies [31,36]. Our study demonstrated that this conventional treatment remained effective during the first week after treatment, adding supplementary protection to the community. However, the residual insecticide effect was lacking one month later, with a percent control below zero. The lack of residual effect may be explained by the reinvasion of untreated rats, while insecticide powder was scattered progressively, being taken of on feet/shoes and by house cleaning. In addition, if pyrethroid and carbamate insecticides remain effective for 2–4 months, organophosphates have a shorter period of effectiveness, especially in humid conditions [13]. A single insecticide dusting is adequate to lower rat flea burden during an epidemic, but unfortunately, may not be suitable for long term prevention of plague during the transmission season.

### Effectiveness of bait stations and potential use as preventive vector control tool against rat fleas

Results of this study demonstrated that bait station with non-poisonous rodent bait was insufficient to significantly reduce the rat flea burden at least during the first week after their utilization. Caution should be taken when implementing this method, when targeting rat and flea elimination simultaneously [23], otherwise raising the concern of releasing potentially infected host-seeking fleas. Notwithstanding their multiple benefits when compared to direct dusting, the bait box or bait station technique used during this study produced less satisfactory results on reducing rodent flea density. However, some factors may be considered, which may explain the failure of bait stations to attain an effective flea control.

One reason may be a lack of coverage, more likely at the room level than the house level. A study demonstrated that the success of the insecticide delivery tube technique targeting fleas harbored by commensal rat depended on the placement of the device directly on rat runway, in a single room household [37]. In addition, monitoring rat movement in a single-room household scale probably facilitated the implementation of the technique, conversely to multiple-story house. Although, in our study scheme, increasing the bait station number to three per household (one per story) was expected to induce more controlling effect on fleas. An immediate effect was lacking in the three-bait station group, even though the density of bait station per hamlet was three-fold higher. Rats could have easily avoided passing through the wooden tunnel bait station, by modifying their trajectory. It was reported that rats can modify their usual movement pattern to avoid new objects in their environment [38]. With ground dusting method, where almost all sides of room were treated, rodents were constantly in contact with the insecticide dust, explaining the better success of this method when compared to single bait station per room.

Increasing the number of bait station per room can be proposed as solution. However, one challenge limiting the deployment of higher number of bait station was its transportation. The wooden bait station used in this study was bulky and difficult to transport. A new bait station design, made with lighter material and collapsible (like Sherman traps) can resolve the transportation problem, knowing that plague cases in Madagascar often occur in isolated countryside, and are difficult to reach by ground transportation. The width of the bait station can also be reduced, lowering the amount of required insecticide per box, while the number per household can be increased.

When used over six consecutive nights rather than only two, better percent control was obtained with bait station (Table 1, 2017). However, bait stations were constantly visited by rodents at the same rate, from the first night of implementation to the last night. (S1 Table). This observation was consistent with a previous study where the treatment devices were visited equally over the study [37]. If the bait stations were constantly visited, the observation of tracks or bait consumption could not generate important information related to the percentage of resident rodent actually visiting the bait station per night. The efficacy of the bait stations may be jeopardized if only a low proportion of resident rodents were using the bait stations. The abundance of stored crops during the study could have influenced the attractiveness of the bait. If the bait (tomato slice) was entirely consumed by a single rodent, the bait station will be less attractive to the others. This may explain the cumulative efficacy observed through time, when greater percent control was observed after six consecutive nights of utilization rather than after two nights. A solution can be the use of new bait station design, where the bait will be enclosed in compartment inaccessible to rodent but still attractive (wire mesh for example). A durable bait such as peanut butter pellets can be used [27], without the need of replenishment. This improvement will also reduce the cost of the method directly linked to frequent bait replacement and the recruitment of local workers performing daily replacement of the baits.

The potential usage of bait station as a long-term preventive flea control tool should be explored. Lowering the rat flea burden before the onset of plague transmission season can be achieved, provided that fully operational bait stations are maintained in households. In that perspective, the improvement of the bait station design proposed here will improve long term maintenance of the bait station in the community. In addition, the attractiveness of other available bait sources should be considered along with better understanding of rodent species movement and behavior at room level, in order to implement more adapted vector control strategies.

### Effect of insecticide treatment on outdoor rodent fleas

The present study was the first that monitored the impact of indoor insecticide treatment on outdoor-caught rodent fleas. It was reported that rodents passing across bait stations, as well as treated surfaces, can spread insecticide dust in their nest [18]. Unfortunately, this indirect treatment effect was not observed (Fig 3) even though a clear rat movement between outdoor and indoor environments has been described in Madagascar [27]. It is now assumed that indoor interventions have no effect on outdoor rodents, whereas rodent to human flea-borne transmission of plague may occur in both environments [22,39]. A vector control approach targeting outdoor rodent-associated fleas, at least within immediate peri-domestic area, should be implemented in Madagascar. Targeting outdoor rodents and their fleas is more challenging mainly due to difficulties in locating burrows [1,20]. Thanks to the technique initially developed by Leo Kartman [20], the bait station has been the more suitable technique for wild rodent ectoparasite control. Since then, different approaches using bait stations have been developed, according to the targeted species [16,19,21,37,40]. The effectiveness of the bait station type used during study as an outdoor vector control strategy should be addressed, as well as other approaches using systemic insecticides [41–44].

### Control strategy against house fleas

Although insecticide treatments during plague outbreaks are not intended for house fleas, the abundance of *P*. *irritans* has been found associated with high plague risk areas [28]. This study is the first to address the impact of insecticide treatment deployed during plague control on house fleas in Madagascar. High *P*. *irritans* infestation was observed in hamlets across this study, independently of insecticide treatments (Fig 4). Besides, the record of off-host rat fleas (*X*. *cheopis* and *S*. *fonquerniei*) captured amongst house flea is striking, highlighting the risk of plague transmission. However, neither insecticide dusting nor bait stations appeared to be suitable to treat house flea infestation. Significant reduction of house fleas per household was only found 30 days post-treatment, in the insecticide dusted group, but only in two of four hamlets (Fig 4). This delayed effect is not compatible with timeliness required for plague intervention [39]. Although it was not demonstrated during this study, individual household conditions related to general domestic hygiene, insecticide use, presence of domestic animals, insolation, and floor and wall type may play a role in the very heterogenous pattern of house flea abundance in some hamlets (Fig 4) [45]. This may impact the success of insecticide interventions. The WHO recommended the use of a residual spray formulation (instead of dust) to control *P*. *irritans*, targeting more surfaces, by applying it directly onto clothing and bedding in the sleeping area as well as in soil and wall crevices [12–14]. Besides, effective treatment should be supplemented with adequate household hygiene [46], which was insufficient or lacking in a large portion of relatively poor households visited during the study (S1D Fig). Sanitation improvement appeared essential to control of house fleas. Moreover, better knowledge of *P*. *irritans* behavior and ecology can be a great help on its management and the better understanding of plague transmission. Even if *P*. *irritans* was reported to be naturally less susceptible to insecticide than *X*. *cheopis* [47], the development of insecticide resistance in *P*. *irritans* populations can be a serious issue for the control of the species. *P*. *irritans* developing resistance to organochlorines insecticides were already described in frequently treated plague focus [47, 48], as well as the mutation conferring the resistance to pyrethroid insecticides [49]. Unfortunately, data regarding the susceptibility of this species to insecticide are still lacking in Madagascar.

To summarize, this study provided information on the relative effectiveness of vector control tools used for plague containment in Madagascar. It was therefore established that insecticide ground dusting is the most efficient tool to control rodent fleas during epidemics. However, more adapted strategies should be implemented regarding house flea infestation and outdoor rat fleas.

Regarding the advantages offered by using the bait station technique, studies should be continued in order to address the challenges encountered during this study. Recommendations were given for possible improvement regarding technical and operational aspects of bait station technique. In addition, its potential as a preventive vector control tool should be assessed further. Considering the unsatisfactory results obtained during this study, it is not recommended to use the bait stations as described, as principal vector control strategy during plague outbreaks.

## Supporting information

S1 Fig Photos of typical landscape and aspects of hamlets (A and B), housing style (B and C) and the interior of a household kitchen (D) in the study areas.
(TIF)

S2 Fig Photo of the wooden tunnel bait station (Boîte de Kartman) used during the study. (TIF)

S1 Table Daily bait station survey data. Each bait station was checked once a day.
(DOCX)

## References

[1] Dennis DT, Gage KL, Gratz N, Poland JD, Tikhomirov E. Plague Manual : Epidemiology, distribution, surveillance and control World Heal Organ Geneva Switz. Geneva, Switzerland: WHO; 1999;32: 135–165. 10.1017/S1026881200147841

[2] Poland JD, Dennis D. Diagnosis and clinical manifestation. Plague Manual: Epidemiology, Distribution, Surveillance and Control—World Health Organization. Geneva, Switzerland; 1999 pp. 43–53.

[3] Rubini M, Gualdi-Russo E, Manzon VSV, Rinaldo N, Bianucci R. Mortality risk factors show similar trends in modern and historic populations exposed to plague. J Infect Dev Ctries. 2016;10: 488–493. 10.3855/jidc.7974 27249524

[4] Bertherat E. Plague around the world, 2010–2015. WHO Wkly Epidemiol Rec. 2016; Available: http://apps.who.int/iris/bitstream/handle/10665/254296/WER9108_89-93.pdf?sequence=1&isAllowed=y

[5] Andrianaivoarimanana V, Piola P, Wagner DM, Rakotomanana F, Maheriniaina V, Andrianalimanana S, et al Trends of Human Plague, Madagascar, 1998–2016. Emerg Infect Dis. 2019;25: 220–228. 10.3201/eid2502.171974 30666930 PMC6346457

[6] Burki T. Plague in Madagascar. Lancet Infect Dis. Elsevier; 2017;17: 1241 10.1016/S1473-3099(17)30647-3 29173885

[7] Tsuzuki S, Lee H, Miura F, Chan YH, Jung S-M, Akhmetzhanov AR, et al Dynamics of the pneumonic plague epidemic in Madagascar, August to October 2017. Euro Surveill. European Centre for Disease Prevention and Control; 2017;22 10.2807/1560-7917.ES.2017.22.46.17-00710 PMC5718396 29162211

[8] Duplantier J, Duchemin J. From the recent lessons of the Malagasy foci towards a global understanding of the factors involved in plague reemergence. Vet Res. 2005; Available: http://www.vetres.org/articles/vetres/abs/2005/03/v4060/v4060.html 10.1051/vetres:2005007 15845233

[9] Miarinjara A, Rogier C, Harimalala M, Ramihangihajason TR, Boyer S. Xenopsylla brasiliensis fleas in plague focus areas, Madagascar. Emerg Infect Dis. 2016;22. doi:cited]. 10.3201/eid2212.160318 PMC5189135 27513742

[10] Ratovonjato J, Rajerison M, Rahelinirina S, Boyer S. Yersinia pestis in Pulex irritans fleas during plague outbreak, Madagascar. Emerg Infect Dis. 2014;20: 1414–1415. Available: http://www.ncbi.nlm.nih.gov/pmc/articles/PMC4111190/ 10.3201/eid2008.130629 25061697 PMC4111190

[11] Gratz NG. Control of plague transmission Plague manual: epidemiology, distribution, surveillance and control. Geneva, Switzerland: World Health Organization; 1999 pp. 101–179.

[12] Rozendaal JA. Vector Control, Methods for Use by Individual and Communities WHO, Geneva 1997.

[13] World Health Organization. Pesticides and their application for the control of vectors and pets of public health importance [Internet]. Sixth edit. C.F. Curtis (London School of Hygiène and Tropical Medecine), editor. Geneva, Switzerland: Department of Control of Neglected Tropical Diseases, WHO Pesticide Evaluation Scheme (WHOPES); 2006. Available: http://apps.who.int/iris/handle/10665/69223

[14] WHO. Operational guidelines on plague surveillance, diagnosis, prevention and control [Internet]. New Delhi: Regional Office for South-East Asia; 2009. Available: https://scholar.google.fr/scholar?q=Operational+Guidelines+on+Plague+Surveillance%2C+Diagnosis%2C+Prevention+and+Control&btnG=&hl=fr&as_sdt=0%2C5#0

[15] Fenske RA, Black KG, Elkner KP, Lee CL, Methner MM, Soto R. Potential exposure and health risks of infants following indoor residential pesticide applications. Am J Public Health. 1990;80: 689–693. 10.2105/ajph.80.6.689 1693041 PMC1404741

[16] Gage KL, Maupin GO, Montenieri J, Piesman J, Dolan M, Panella NA. Flea (Siphonaptera: Ceratophyllidae, Hystrichopsyllidae) and Tick (Acarina: Ixodidae) Control on Wood Rats Using Host-Targeted Liquid Permethrin in Bait Tubes. J Med Entomol. 1997;34: 46–51. 10.1093/jmedent/34.1.46 9086710

[17] Kartman L. Further observations on an insecticide-bait-box method for the control of sylvatic plague vectors; effect of prolonged field exposure to DDT powder. J Hyg (Lond). 1960; Available: http://journals.cambridge.org/abstract_S0022172400038171

[18] Kartman L. An insecticide-bait-box method for the control of sylvatic plague vectors. J Hyg (Lond). 1958;56: 455–465. 10.1017/S0022172400037967 13611242 PMC2218086

[19] Barnes A, Kartman L. Control of plague vectors on diurnal rodents in the Sierra Nevada of California by use of insecticide bait-boxes. J Hyg (Lond). 1960;58: 347–355. Available: http://journals.cambridge.org/abstract_S0022172400038468 13687091 10.1017/s0022172400038468 PMC2134376

[20] Kartman L, Lonergan R. Wild-rodent-flea control in rural areas of an enzootic plague region in Hawaii: A preliminary investigation of methods. Bull World Health Organ. 1955;13: 49 Available: http://www.ncbi.nlm.nih.gov/pmc/articles/PMC2538036/ 13260882 PMC2538036

[21] Lane RS, Casher LE, Peavey CA, Piesman J. Modified bait tube controls disease-carrying ticks and fleas. Calif Agric. 1998;52: 43–48. 10.3733/ca.v052n02p43

[22] Migliani R, Chanteau S. Epidemiological trends for human plague in Madagascar during the second half of the 20th century: a survey of 20 900 notified cases. Trop Med Int Heal. 2006;11: 1228–1237. Available: http://onlinelibrary.wiley.com/doi/10.1111/j.1365-3156.2006.01677.x/full 10.1111/j.1365-3156.2006.01677.x 16903886

[23] Ratovonjato J, Duchemin JB, Duplantier JM, Rahelinirina S, Soares JL, Rahalison L, et al Lutte contre la peste à Madagascar : évaluation de l’efficacité des boîtes de Kartman en milieu urbain. Arch Inst Pasteur Madagascar.; 2003;69: 41–45. Available: http://www.pasteur.mg/wp-content/uploads/2016/04/Archives-Institut-Pasteur-de-Madagascar-2003-69-41-45.pdf 15678815

[24] Rakotoarisoa A, Ramihangihajason T, Ramarokoto C, Rahelinirina S, Halm A, Piola P, et al Bubonic Plague Outbreak Investigation in the Endemic District of Tsiroanomandidy—Madagascar, October 2014. J Cases Rep Stud. 2016;5: 1–6.

[25] Borchert JN, Davis RM, Poché RM. Field efficacy of rodent bait containing the systemic insecticide imidacloprid against the fleas of California ground squirrels. J Vector Ecol. Wiley/Blackwell (10.1111); 2009;34: 92–98. 10.1111/j.1948-7134.2009.00011.x 20836808

[26] Rahelinirina S, Duplantier J-M, Ratsimba M, Ratovonjato J, Ramilijaona O, Papillon Y, et al Assessment of Rhodamine B for labelling the plague reservoir Rattus rattus in Madagascar. Afr J Ecol. 2009; 10.1111/j.1365-2028.2009.01162.x

[27] Rahelinirina S, Duplantier JM, Ratovonjato J, Ramilijaona O, Ratsimba M, Rahalison L. Study on the movement of Rattus rattus and evaluation of the plague dispersion in Madagascar. Vector Borne Zoonotic Dis. 2010;10: 77–84. 10.1089/vbz.2009.0019 20158335

[28] Laudisoit A, Leirs H, Makundi RH, Van Dongen S, Davis S, Neerinckx S, et al Plague and the human flea, Tanzania. Emerg Infect Dis. 2007;13: 687–693. 10.3201/eid1305.061084 17553245 PMC2738476

[29] Andrianaivoarimanana V, Kreppel K, Elissa N, Duplantier J-MM, Carniel E, Rajerison M, et al Understanding the persistence of plague foci in Madagascar. PLoS Negl Trop Dis. 2013;7: e2382 10.1371/journal.pntd.0002382 24244760 PMC3820717

[30] Kilonzo BS. A simple light trap for field collection of adult fleas: studies on its efficiency and usability in North-East Tanzania. WHO/VBC/77673 11P. 1977;

[31] Miarinjara A, Boyer S. Current Perspectives on Plague Vector Control in Madagascar: Susceptibility Status of Xenopsylla cheopis to 12 Insecticides. PLoS Negl Trop Dis. Public Library of Science; 2016;10: e0004414 10.1371/journal.pntd.0004414 26844772 PMC4742273

[32] RStudio: integrated development environnement for R RStudio Inc. Boston, Massasuchetts, USA: RStudio Team; 2018.

[33] Henderson CF, Tilton EW. Tests with Acaricides against the Brown Wheat Mite. J Econ Entomol. 1955;48: 157–161. 10.1093/jee/48.2.157

[34] Mount GA, Grothaus RH, Reed JT, Baldwin KF. Amblyomma americanum: Area Control with Granules or Concentrated Sprays of Diazinon, Propoxur, and Chlorpyrifos12. J Econ Entomol. Oxford University Press; 1976;69: 257–259. 10.1093/jee/69.2.257 57127

[35] GraphPad Prism version 7.04 for Windows San Diego, California, USA: GraphPad Software Inc.; 2017.

[36] Miarinjara A, Vergain J, Kavaruganda JM, Rajerison M, Boyer S. Plague risk in vulnerable community: Assessment of Xenopsylla cheopis susceptibility to insecticides in Malagasy prisons. Infect Dis Poverty. 2017;6 10.1186/s40249-017-0356-5 29110719 PMC5674827

[37] Boegler KA, Atiku LA, Mpanga JT, Clark RJ, Delorey MJ, Gage KL, et al Use of Insecticide Delivery Tubes for Controlling Rodent-Associated Fleas in a Plague Endemic Region of West Nile, Uganda. J Med Entomol. 2014;51: 1254–1263. 10.1603/ME14083 26309315 PMC4599340

[38] Brooks J.E. and Rowe F.P. Commensal rodent control. Who/Vbc/79/726,. 1987.

[39] Boegler KA, Atiku LA, Enscore RE, Apangu T, Mpanga JT, Acayo S, et al Rat fall surveillance coupled with vector control and community education as a plague prevention strategy in the West Nile Region, Uganda. Am J Trop Med Hyg; 2018;98: 238–247. 10.4269/ajtmh.17-0502 29141768 PMC5928726

[40] Bronson LR, Smith CR. Use of liquid deltamethrin in modified, host-targeted bait tubes for control of fleas on sciurid rodents in northern California. J Vector Ecol. 2002;27: 55–62. Available: http://www.ncbi.nlm.nih.gov/pubmed/12125873 12125873

[41] Rood MP, Poché RM. Effect of a Feed-Through Insecticide (Imidacloprid) on the Flea Index of a Norway Rat (Rattus norvegicus) Focus in Los Angeles, California. Procedings of 24th Vertebrate Pest Conference. 2010.

[42] Poché DM, Hartman D, Polyakova L, Poché RM. Efficacy of a fipronil bait in reducing the number of fleas (Oropsylla spp.) infesting wild black-tailed prairie dogs. J Vector Ecol. 2017;42: 171–177. 10.1111/jvec.12252 28504448

[43] Poché DM, Torres-Poché Z, Yeszhanov A, Poché RM, Belyaev A, Dvořák V, et al Field evaluation of a 0.005% fipronil bait, orally administered to Rhombomys opimus, for control of fleas (Siphonaptera: Pulicidae) and phlebotomine sand flies (Diptera: Psychodidae) in the Central Asian Republic of Kazakhstan. PLoS Negl Trop Dis. 2018;12: 1–23. 10.1371/journal.pntd.0006630 30044788 PMC6059381

[44] Rajohnson D, Miarinjara A, Rahelinirina S, Boyer S. Effectiveness of fipronil as a systemic control agent against Xenopsylla cheopis (Siphonaptera: Pulicidae) in Madagascar. J Med Entomol. 2017;54: 1–7. 10.1093/jme/tjw200 28122816

[45] Yin J-X, Geater A, Chongsuvivatwong V, Dong X-Q, Du C-H, Zhong Y-H. Predictors for abundance of host flea and floor flea in households of villages with endemic commensal rodent plague, Yunnan Province, China. PLoS Negl Trop Dis. 2011;5: e997 10.1371/journal.pntd.0000997 21468306 PMC3066137

[46] Degroote S, Zinszer K, Ridde V. Interventions for vector-borne diseases focused on housing and hygiene in urban areas: a scoping review. Infect Dis poverty. BioMed Central; 2018;7: 96 10.1186/s40249-018-0477-5 30173670 PMC6120073

[47] Smith A. The susceptibility to dieldrin of Pulex irritans and Pediculus humanus corporis in the Pare area of north-east Tanganyika. Bull World Health Organ. 1959;21: 240–241 13831868 PMC2537873

[48] Karimi Y, Eftekhari M, Almeida CR. Sur L’écologie des puces impliquées das L’épidemiologie de la peste et le rôle éventuel de certains insectes hématophages dans son processus au nord-est du Brésil. Bull Soc Pathol Exot. 1974 pp. 583–591. 4480536

[49] Ghavami MB, Haghi FP, Alibabaei Z, Enayati AA, Vatandoost H. First report of target site insensitivity to pyrethroids in human flea, Pulex irritans (Siphonaptera: Pulicidae). Pestic Biochem Physiol. Academic Press; 2018;146: 97–105. 10.1016/j.pestbp.2018.03.004 29626998

