# Supplementary figures and images for: Field assessment of insecticide dusting and bait station treatment impact against rodent flea and house flea species in the Madagascar plague context
Source: PLoS Negl Trop Dis. 2019 Aug 6;13(8):e0007604. doi: 10.1371/journal.pntd.0007604 (PMC6697362; doi:10.1371/journal.pntd.0007604)

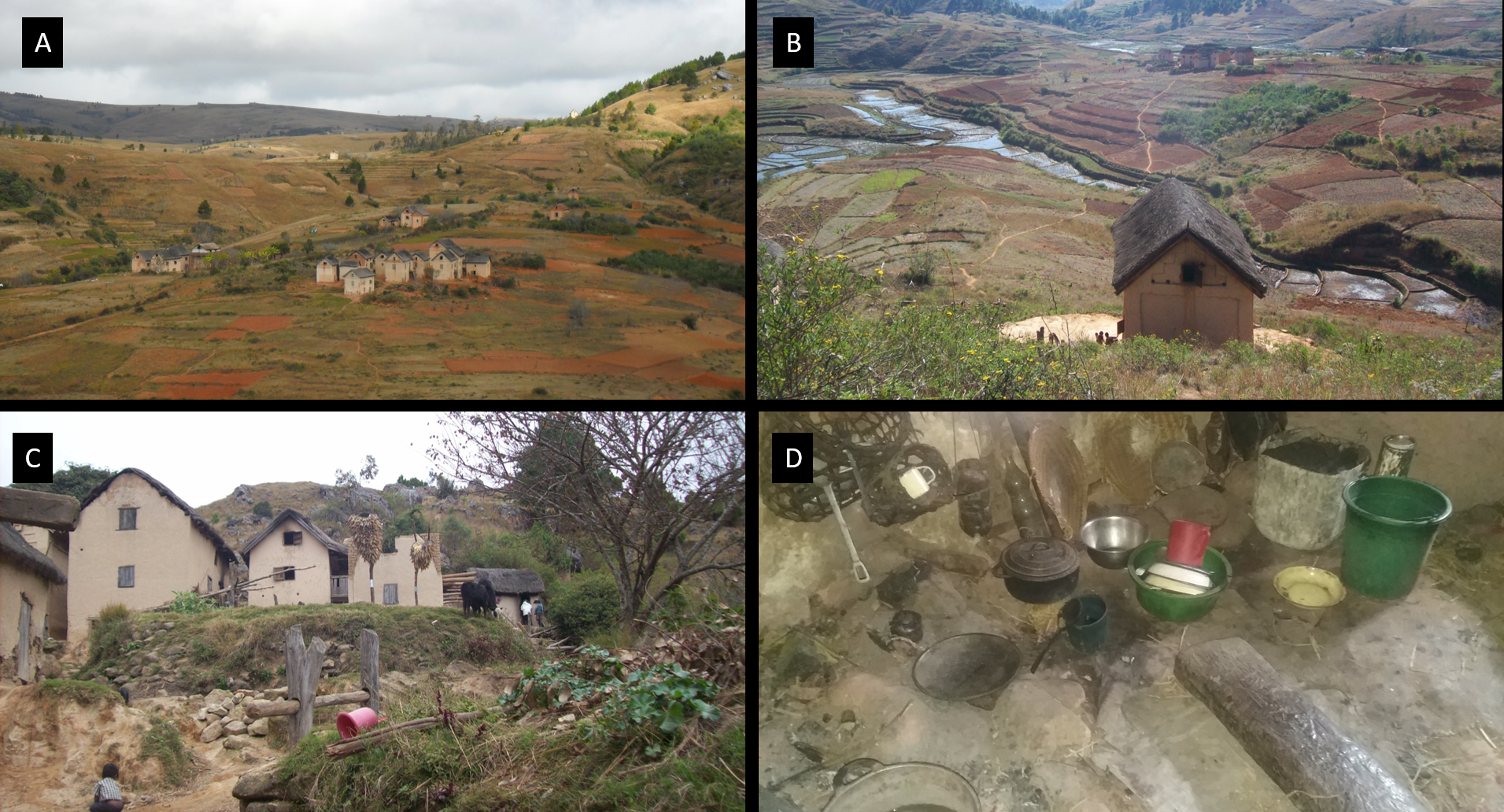

Supplement: S1 Fig — Photos of typical landscape and aspects of hamlets (A and B), housing style (B and C) and the interior of a household kitchen (D) in the study areas. (TIF) [file pntd.0007604.s001.tif]

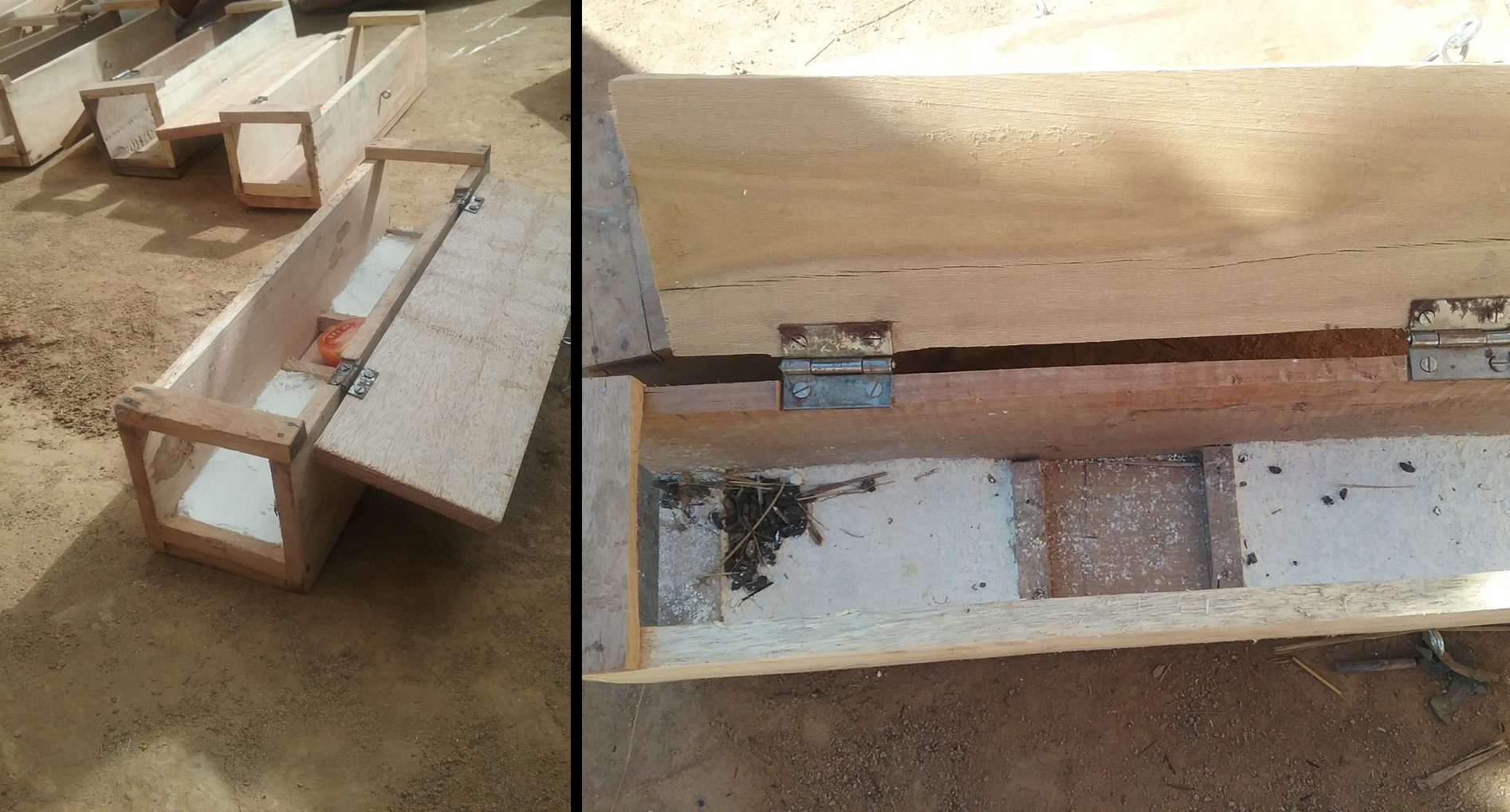

Supplement: S2 Fig — (TIF) [file pntd.0007604.s002.tif]
