# Supplementary material for: Field assessment of insecticide dusting and bait station treatment impact against rodent flea and house flea species in the Madagascar plague context
Source: PLoS Negl Trop Dis. 2019 Aug 6;13(8):e0007604. doi: 10.1371/journal.pntd.0007604 (PMC6697362; doi:10.1371/journal.pntd.0007604)
Supplement: S1 Table — Each bait station was checked once a day. (DOCX) [file pntd.0007604.s003.docx]

|  | One bait station per household | | | Three bait stations per household | | |
| --- | --- | --- | --- | --- | --- | --- |
|  | Bait station number | Visited ^a^ (%) | Bait consumed ^b^ (%) | Bait station number | Visited ^a^ (%) | Bait consumed ^b^ (%) |
| Night 1 | 59 | 91.53 | 69.49 | 174 | 71.84 | 61.49 |
| Night 2 | 59 | 83.05 | 81.36 | 174 | 84.48 | 85.63 |
| Night 3 | 59 | 86.44 | 72.88 | 171 | 83.04 | 81.29 |
| Night 4 | 59 | 91.53 | 79.66 | _ | _ | _ |
| Night 5 | 59 | 89.83 | 83.05 | _ | _ | _ |
| Night 6 | 59 | 72.88 | 62.71 | _ | _ | _ |
| ^a^ Percent bait station where rodent activities were observed (track only or/and nibbled or totally consumed bait). ^b^ Percent bait station where baits were nibbled or totally consumed. | | | | | | |
